# Ecotypic variation in growth responses to simulated herbivory: trade-off between maximum relative growth rate and tolerance to defoliation in an annual plant

**DOI:** 10.1093/aobpla/plv015

**Published:** 2015-02-27

**Authors:** Iván D. Camargo, Rosalinda Tapia-López, Juan Núñez-Farfán

**Affiliations:** 1 Departamento de Ecología Evolutiva, Instituto de Ecología, Posgrado en Ciencias Biológicas, Universidad Nacional Autónoma de México, 04510 México, Distrito Federal, México; 2 Present address: Universidad Autónoma de Guadalajara, Campus Tabasco, CP 86280, Villahermosa, México

**Keywords:** *Datura stramonium*, defoliation tolerance, growth response coefficients, net assimilation rate, ontogenetic plasticity response to defoliation, plant relative growth rate

## Abstract

It has been hypothesized that slow-growing plants are more likely to maximize above-ground biomass and fitness when defoliated by herbivores than those with an already high relative growth rate (RGR). Some populations of the annual herb *Datura stramonium* L. can tolerate foliar damage better than others. The physiological basis of this difference is examined here in a comparative study of two ecotypes that differ in tolerance and maximum growth rate, using a growth analytical approach. One hundred and fifty-four plants of each ecotype grown under controlled conditions were suddenly defoliated (35 % of total leaf area removed) and a similar sample size of plants remained undefoliated (control). Ontogenetic plastic changes in RGR and its growth components [net assimilation rate (NAR), specific leaf area and leaf weight ratio (LWR)] after defoliation were measured to determine whether these plastic changes maximize plant growth and fitness. Different ontogenetic phases of the response were discerned and increased RGR of defoliated plants was detected at the end of the experimental period, but brought about by a different growth component (NAR or LWR) in each ecotype. These changes in RGR are putatively related to increases in fitness in defoliated environments. At the intra-specific scale, data showed a trade-off between the ability to grow under benign environmental conditions and the ability to tolerate resource limitation due to defoliation.

## Abstract (precis)

How fast plant genotypes grow before damage by herbivores has been theorized to impact negatively on the tolerance response to defoliation. Using a growth analytical approach in two ecotypes of *Datura stramonium* that differ in relative growth rate (before defoliation) and tolerance to defoliation, this study shows that slow growing plant genotypes exhibit the highest compensation (more trait expression) after defoliation, not only in relative growth rate, but also in fitness (number of seeds). At the intra-specific scale, a trade-off between the ability to grow under benign environmental conditions and the ability to tolerate resource limitation due to defoliation was detected.

## Introduction

As sessile organisms, plants must cope with biotic and abiotic spatio-temporal environmental fluctuations by means of phenotypic plasticity (Schlichting 1986). Although plants are commonly defoliated by herbivores, they may mitigate their negative effects through compensatory growth (McNaughton 1983). Compensatory response implies a series of plastic phenotypic changes in different traits that can determine the pattern of change in relative growth rate (RGR, the rate at which a given amount of existing biomass can produce new biomass per unit time; Shipley 2000) after defoliation (Oesterheld and McNaughton 1988; Trumble *et al.* 1993; Strauss and Agrawal 1999; Stowe *et al.* 2000; Tiffin 2000). These plastic responses to defoliation may include an increasing photosynthetic rate (Caldwell *et al.* 1981; Wallace *et al.* 1984), changing the allocation pattern to increase the production of new leaf area (McNaughton and Chapin 1985), or nutrient uptake (Ruess *et al*. 1983; McNaughton and Chapin 1985) and improving plant water status (Toft *et al.* 1987).

The interplay between these partial plastic traits determines the plant’s overall response to defoliation, which may contribute to tolerance alleviating negative effects on fitness (McNaughton 1983; Núñez-Farfán *et al*. 2007). A genotype is considered more tolerant when defoliation has a lower effect on fitness, even to a point at which it may seem that it is not perceiving the stress or overcoming it (i.e. overtolerance, higher fitness in the defoliated environment). Such apparent lack of effect on fitness could be controlled by plastic changes in morphological and physiological traits (Bradshaw 1965; Richards *et al*. 2006; Sultan *et al.* 1998), which are less visible and more distant to fitness (i.e. underlying traits *sensu* Alpert and Simms 2002). In other words, defoliation can modify underlying traits that maintain fitness stability and the stress could be perceived at the ‘macroscopic’ level, affecting fitness or sometimes only at a more detailed level, modifying underlying traits (Couso and Fernández 2012). Thus, the degree and pattern of plasticity in tolerance traits could result in three different patterns of plant fitness between undefoliated and defoliated environments; namely, complete tolerance (no fitness differences between environments), undertolerance (lower fitness in the defoliated environment) and overtolerance (higher fitness in the defoliated environment). Many species fail to compensate after defoliation and are presumed to be undertolerant (Bergelson *et al*. 1996; Stowe 1998; Hanley and Fegan 2007; Klimešová *et al.* 2007), while a full range of compensatory responses has been observed for complete tolerance and overtolerance (Paige and Whitham 1987; Lennartsson *et al.* 1998; Hochwender *et al.* 2000; Hawkes and Sullivan 2001).

According to Hilbert *et al*. (1981) there is a trade-off between the ability of a genotype to grow under undefoliated environmental conditions (i.e. the environment at which is achieved the maximum growth potential, RGR_max_), and its capacity to tolerate defoliation. They specifically predict that plants growing at nearly their shoot RGR_max_ were less likely to maximize above-ground biomass if defoliated than those with shoot growth rates far below maximum. This is because genotypes with high RGR_max_ at the time of grazing require large increases in growth rate while slow growth genotypes require only small increases to reach undefoliated plants (Hilbert *et al*. 1981). Furthermore, after defoliation, genotypes with slow RGR_max_ exhibit the highest overcompensatory growth later (higher RGR in defoliated plants) (Oesterheld and McNaughton 1988).

Compensatory growth constitutes one of the most important traits related to defoliation tolerance, because in annuals it is positively related, via attainment of a larger size, with reproductive effort (Weaver and Cavers 1980; Aarssen and Taylor 1992), survival and fecundity (Crawley 1997). Most studies on compensatory growth have concluded that, to compensate for biomass lost, a defoliated plant must have a higher RGR than an undefoliated plant (i.e. a higher rate of increasing biomass per unit of biomass already present) (Hilbert *et al*. 1981). This increase in RGR is the result of comparisons often made at a single fixed point in time after defoliation (Oesterheld and McNaughton 1991); however, environmental differences in RGR are the result of plasticity in developmental trajectories (i.e. ontogenetic plasticity; Pigliucci 2001). Whether RGR increase in defoliated plants is sufficient to produce as much biomass as the undefoliated plants would depend on how long that environmental difference is maintained (Hilbert *et al.* 1981). Therefore, monitoring the growth difference of defoliated and undefoliated plants over time may be of great value for the study of the response to defoliation (Hilbert *et al.* 1981; Oesterheld and McNaughton 1991). This approach has proved successful in the study of other environmental plant stressors (Shipley 2000; Useche and Shipley 2010*a*, *b*). However, little is known about the time-course of growth, and frequently it is ignored or assumed to be monotonic in defoliated environments (but see Oesterheld and McNaughton 1988).

Plant growth analysis can be used to assess the contribution of different mechanisms of compensatory growth to RGR via its three determinants. First, the net assimilation rate (NAR)—the increase in biomass per unit of time and leaf area—is strongly correlated with the whole-plant net photosynthetic rate (Poorter and Van der Werf 1998). Second, specific leaf area (SLA, leaf area per unit of leaf biomass) is a parameter that reflects aspects of leaf morphology such as leaf density and thickness (Poorter and Nagel 2000). And third, the biomass allocation to leaves (LWR) needs to be considered. Thus, RGR can be broken down into these three leaf-based properties as follows: RGR = NAR × SLA × LWR. The product of SLA and LWR is the leaf area ratio (i.e. LAR, the ratio of leaf area per plant biomass). To quantify the degree to which plants can compensate for potential losses in RGR throughout development due to defoliation, we asked to what extent a difference in RGR caused by a difference in resource supply due to defoliation (in the case of defoliation, decreases in resource supplies such as light and CO_2_ are expected; Trumble *et al.* 1993) is due to a difference in each of the growth determinants. In other words, how do the different components change if RGR changes due to defoliation? In order to answer this question we used a plasticity estimation called the Growth Response Coefficient (GRC; Poorter and Van der Werf 1998): the relative change in one of the growth determinants, scaled with respect to the relative change in RGR (Poorter and Nagel 2000; see Methods section) due to defoliation.

**Figure 1.**
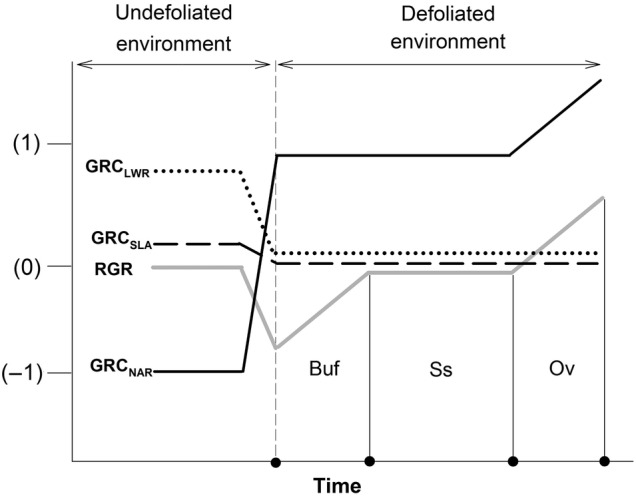
Hypothetical scenario relating plastic changes in RGR (signed percentages of increase or decrease of defoliated plants in relation to control plants) and the relative contribution (i.e. a GRC) of NAR, SLA and LWR to these plastic changes in a plant whose leaf-total area is highly defoliated. By definition, before defoliation (dashed line reference), the plasticity in RGR is zero. Therefore, the trajectory of RGR before defoliation is not shown, but it decreases in the model due to the negative influence of NAR. The plastic behaviour of RGR has three main ontogenetic stages (dots and vertical lines in the time axis) in the model (Oesterheld and McNaughton 1988, 1991): a buffering state (Buf), steady state (Ss) and an overcompensatory state (Ov) (see Introduction). The range of GRC values indicates the severity of a given reduction in resource supply due to defoliation (see Methods). In the model NAR plays a leading role in the RGR's buffering and Ov.

Time-course changes in plant growth and allocation parameters following a defoliation event have been reported (Oesterheld and McNaughton 1988; Anten *et al.* 2003; Van Staalduinen and Anten 2005). However, there is no consensus on how plants are expected to respond after biomass removal (Yoshizuka and Roach 2011). In response to defoliation, the behaviour of RGR suffers an oscillation over time (Oesterheld and McNaughton 1988, 1991) that can be classified into three main ontogenetic stages: initially decreasing after defoliation but later recovering to the values of control plants (buffering state); a relatively unchanging time behaviour with respect to control plants (steady state), but later reaching values above those of control plants (overcompensatory state). The buffering capacity of RGR has been related to strong progressive ontogenetic increases in NAR, and SLA counteracting the immediate decrease in LWR after defoliation, to reach the same LAR (new leaf area at a low carbon cost) as control plants (Oesterheld and McNaughton 1988, 1991). While this counteractive action of the growth components has been related to the buffering capacity of a plant's RGR, instances of steady and overcompensatory growth have received less attention (Fig. 1).

Here, we studied the amount and timing of ontogenetic plastic changes in RGR and its determinants following a single defoliation in *Datura stramonium* L. (Solanaceae), a colonizing annual plant distributed world-wide. We compared two ecotypes of *D. stramonium* with differences in tolerance to defoliation, associated with differences in herbivore pressure in different abiotic environments (Fornoni *et al.* 2003, 2004). Since there is a great variation in the response to herbivory at the intra-specific level in *D. stramonium*, the comparisons of growth parameters of defoliated and undefoliated plants of different ecotypes would provide insights into the different defensive strategies that plants evolve in interaction with herbivores (e.g. Oesterheld and McNaughton 1988).

The main hypothesis is that there is a trade-off between tolerance to defoliation and growth under less limiting conditions (undefoliated environments), but that this model only cover traits related directly to fitness, i.e. those at the macroscopic level (e.g. above-ground biomass and the number of seeds). The secondary hypothesis is, at a more detailed level here, that the more tolerant genotypes are more plastic in mechanistic traits (i.e. compensatory growth), those that would allow them to gain more fitness in the defoliated environment.

## Methods

*Datura stramonium* is a self-compatible annual weed occurring in a wide variety of plant communities in Mexico and North America (Weaver and Warwick 1984). Although it is found in all types of soil, it prefers rich soils (Weaver and Warwick 1984) and rapidly assimilates nitrogen in the form of nitrate or ammonium (Lewis and Probyn 1978; Platt and Rand 1982). Its leaves are eaten by at least two specialist herbivorous insects, *Epitrix parvula* (Coleoptera: Chrysomelidae) (Núñez-Farfán and Dirzo 1994) and *Lema trilineata* (Coleoptera: Chrysomelidae), and other generalist species (Núñez-Farfán and Dirzo 1994; Núñez-Farfán *et al.* 1996). The average percentage of leaf area lost to herbivores in populations of *D. stramonium* from Central Mexico is 30 % (range 11–49 %; Valverde *et al.* 2001). In *D. stramonium*, unlike many plants where the adult phase begins when sexuality is first expressed, maturity and sexuality are not necessarily synonymous, as sexuality is also very important for the vegetative growth dynamics of the plant due to its particular architectural model called *Leeuwenberg* (Hallé *et al*. 1978), in which branching produces equivalent orthotropic modules, each with deterministic growth, culminating in the production of a terminal flower.

### Growth conditions and harvest

Seeds from two populations (natural progenies) of *D. stramonium* from Central Mexico were collected from the Santo Domingo (SD) population in the state of Morelos (18°N, 99°W) and the Patria Nueva (PN) population in the state of Hidalgo (20°N, 99°W). Localities differ in climate, vegetation and type of soil (Table 1). Plants of both populations withstand different natural levels of defoliation by herbivores, with a difference in abiotic pressure perhaps contributing to select different growth components of RGR in response to defoliation (Poorter and Nagel 2000). Santo Domingo population has shown to be overtolerant to natural defoliation (e.g. Fornoni *et al*. 2003) while PN population to be complete tolerant (e.g. Valverde *et al*. 2001).

**Table 1.** Environmental characteristics and mean relative resistance (1-natural defoliation) of two *D. stramonium* populations in Central Mexico. ^1^Standard error, *n*.

Characteristics	*D. stramonium* populations		Sources
	Santo Domingo	Patria Nueva	
Habitat	Pine-oak forest	Xerophytic shrub	García (1988)
Geographic coordinates	18°N, 99°W	20°N, 99°W	García (1988)
Altitude (m a.s.l)	2050	1745	García (1988)
Mean annual precipitation (mm)	1463.2	360.5	García (1988)
Mean annual temperature (°C)	19.9	18.4	García (1988)
Relative resistance	0.559 (0.014, 30)^1^	0.816 (0.013, 18)^1^	Valverde *et al*. (2001)

Full-sibs of each field's maternal plant were derived from one generation of selfing at the glasshouse. For germination, seeds were put in pots (1.5 L) and kept at a 12:12 h (light/dark) photoperiod in a Roch growth chamber, and mean temperature of 28/23 °C. When cotyledons were fully expanded, seedlings were transplanted to pots with a mixture of sand and peat moss (4 : 1), and placed at the glasshouse. We discarded half of the plants prior to transplanting to assure a reduction in the variability expressed in seed germination and to ensure that the growth conditions were as similar as possible between populations. The mean radiation was 231 W m^−2^. The temperature regime was 20 ± 0.5 °C day and 13 ± 0.5 °C night (13 h daylength). Relative humidity was always above 60 %. The day before transplanting, the 25 % largest and 25 % smallest plants from each population were discarded (Poorter 1989). Nutrients were added in the form of 200 mL of a liquid soluble fertilizer (Peter's 20–20–20: 3.9 % NH_4_-N, 5.8 % NO_3_-N, 10.0 % urea-N, 20 % P_2_O_5_-P, 20 % K_2_O-K), such that total nutrient addition over the entire growth period approximated to a nutrient supply of 400 kg N ha^−1^year^−1^ (medium nutrient level in Table 1 of McConnaughay and Coleman 1999).

The experiment consisted of 154 plants for each population. One half of the plants experienced no defoliation over the entire experiment. The other half of the plants were defoliated to the nearest 35 % of total leaf area removed. This defoliation level was chosen to simulate the upper limit of natural defoliation intensity observed in the sampled populations (∼31 % of mean leaf area consumed; Table 1). Using the estimated relationship between leaf length (cm) and leaf area (cm^2^) (leaf area = 0.56–0.76[leaf length] + 0.5[leaf length]^2^; *r*^2^ = 0.99), we were able to estimate the area to be removed with a cork-borer No. 6 (1.1 cm^2^ in diameter), when plants were 61 days old (1464 h of age), after seedling emergence. The experiment was run mainly in the adult phase, but in this species sexuality is also very important for the vegetative growth dynamics of the plant; nearly the 60 % of the total vegetative growth is achieved in the adult phase (I. D. Camargo and J. Núñez-Farfán, unpubl. data).

The harvest schedule began at 59 days (1416 h) after seedling emergence and continued for 74.25 days (1782 h). To detect plastic responses to defoliation, the harvest programme was conducted with a more intense harvest frequency, bracketing the day when the treatment (i.e. defoliation) was imposed (e.g. Shipley 2000; Useche and Shipley 2010*a*, *b*). On Day 59, two plants per population per experimental condition were harvested in the morning (09:00), noon (12:00) and in the afternoon (15:00); from Day 60 (i.e. 1 day before defoliation) until Day 64, two plants per population per experimental condition were harvested at 09:00, 11:00, 13:00 and 15:00; from Day 65 until Day 67, two plants per population per experimental condition were harvested at 11:00 and 15:00; and from Day 68 until Day 74, one plant per population per experimental condition was harvested at 15:00. Plants to harvest were randomly chosen.

Plants chosen at each harvest were separated into leaves, ‘support’ tissues (stems, petioles and pedicels) and reproductive tissues (flowers and fruits), if present. Plant parts and their dry weight were measured after being oven-dried for at least 48 h at 80 °C. Total one-side of fresh leaf area of the plant was estimated by image analysis with Compu Eye, Leaf and Symptom Area Software (Bakr 2005).

### Growth analysis and plasticity estimation

Plant traits were measured following Shipley's (2000) protocol. In general, the SLA was calculated as the whole-plant leaf area divided by whole-plant leaf dry mass. The LWR was calculated as the leaf dry mass divided by shoot dry mass. The predicted values across time of leaf area, shoot dry mass, SLA and LWR were evaluated using cubic splines by means of the *gam* function in the R package MGCV (Wood 2011).

The use of peat moss in the soil mixture precluded the estimation of the root mass ratio; however, using only above-ground tissues in the determination of maximum RGR substantially reduces the time and effort required in harvesting plants (Shipley 1989). Notwithstanding, defoliation has a minimal effect on root biomass of grasses and annual herbs that do not form rhizomes (reviewed in Ferraro and Oesterheld 2002) as *D. stramonium*. The RGR was calculated as the rate of change in the natural logarithm of shoot dry mass over time, obtained as the derivative of the cubic-spline smoother using the *smooth spline* function in R (R Foundation for Statistical Computing; http://www.R-project.org). Cubic-spline smoothers have been shown to accurately detect even subtle changes in RGR without imposing any functional assumptions on the data (Shipley and Hunt 1996). The NAR was calculated as RGR divided by the product of SLA and LWR.

All other statistical analyses testing the differences between the control and defoliated environments were done using a General Additive Model as implemented in the R package MGCV (Wood 2011) (see Appendix). We let the smooths ‘interact’ with the experimental conditions as a factor (control and defoliated series) and its significance was interpreted as an ontogenetic plasticity to defoliation in any trait measured. For each experimental condition, we allowed for smooths to have different smoothing parameters. The *P*-values for individual terms were calculated using the Bayesian estimated covariance matrix of the parameter estimators implemented in the MGCV package, based on a test statistic motivated by Nychka's (1988) analysis of the frequentist properties of Bayesian confidence intervals for smoothing splines (Wood 2011). When the treatment effect was significant, standard errors based on the Bayesian posterior covariance of the parameters in the fitted model were used (Wahba 1983; Wood 2011) as a visual aid to interpret the ontogenetic variation of the statistical difference between control and defoliated environments in the plot of any trait measured.

The degree of plasticity for each ontogenetic parameter was estimated as the difference between the defoliated plants and control plants divided by control plants, and therefore it reflects signed percentages of increase or decrease in defoliated plants with respect to controls. The use of signed plasticity values allowed us to evaluate the progressive ontogenetic plastic adjustments of defoliated plants to restore the trajectory of controls and, therefore, to identify whether the plastic response was active, or whether it was passive, from resource deficiency (van Kleunen and Fischer 2005). For instance, an initial decrease in LWR is expected due to defoliation (algebraically, the biomass of leaves decreases in proportion to all biomass present); the progressive recovery of defoliated plants in order to re-attain the trajectory of the controls across time in terms of this parameter is what constitutes an active response to defoliation.

To compare the relative contribution of each growth determinant to the RGR differences observed in response to defoliation, we estimated the GRC (*sensu* Poorter and Nagel 2000) for each growth determinant. Growth response coefficients are scaling (allometric) slopes, in which the natural logarithm of each growth component is regressed on the natural logarithm of RGR (Shipley 2006). Then GRCs for each growth determinant (*X*) were calculated using the differences between defoliated (D) and control plants (C), as follows:

$$\text{GR}{\text{C}}_{X}=\frac{\ln\;X_{D}-\ln\;X_{C}}{\ln\;\text{RG}{\text{R}}_{D}-\ln\;\text{RG}{\text{R}}_{C}}$$

The range of GRC values indicates the severity of a given reduction in resource supply (Poorter and Nagel 2000) due to defoliation. For instance, a GRC value of 1 indicates that the proportional plastic change in the growth parameter of interest equals the proportional plastic change in RGR. A GRC value of 0 indicates that there is no plastic change in that growth parameter at all. Growth response coefficient values can be higher than 1 if the increase in the growth parameter is stronger than the increase in RGR, and can be lower than 0 if an increase in a growth parameter corresponds to a decrease in RGR (Poorter and Nagel 2000). Since the GRCs are proportional to changes in NAR, LWR and SLA relative to RGR, then these values should add up to 1 when RGR is exactly the product of them. Growth response coefficient values before defoliation were estimated as the slope of the linear regression between the natural logarithm of each growth component regressed on the natural logarithm of RGR (Poorter and Van der Werf 1998) in the time interval between 1416 and 1446 h of age. The latter interval is chosen because, by definition, there is no difference (plasticity) between the defoliated and control series in this time interval.

The contribution of ontogenetic values of each GRC to growth were evaluated with a *t*-test (when assumptions were met) or with a Wilcoxon signed-rank test (*T*), using the null hypothesis of GRC*_X_* = 0 (i.e. no contribution to growth). There were two negative predicted RGR values (1734 and 1782 h of age) in the final trajectory of growth in the control series of the SD ecotype; therefore, these were omitted in the estimation and plots of plasticity and GRCs.

#### Tolerance

As a proxy to lifetime reproductive fitness, the final reproductive output was estimated as the total seed number per plant of each ecotype, and measured in an additional sample of 60 plants at the end of the experiment (81 days after germination, 7 days after the last plant growth harvest until the last seeds were mature enough). These plants were not used for the estimation of plant growth traits described above; mainly, because plant leaves at this stage were almost absent by natural defoliation. The slope of fitness between control and defoliated plants was considered as a measure of tolerance to the defoliation. The total seed number was analysed with a general linear model with a Poisson error, and a *log* link function (JMP, Version 7, SAS Institute, Inc., Cary, NC, 1989–2007). This was conducted to investigate the relative importance of (i) ecotype (variation among ecotypes); (ii) treatment (presence of average fitness plasticity in response to defoliation regardless of specific ecotypes); and (iii) treatment by ecotype interaction (variation for fitness plasticity among ecotypes).

## Results

**Figure 2.**
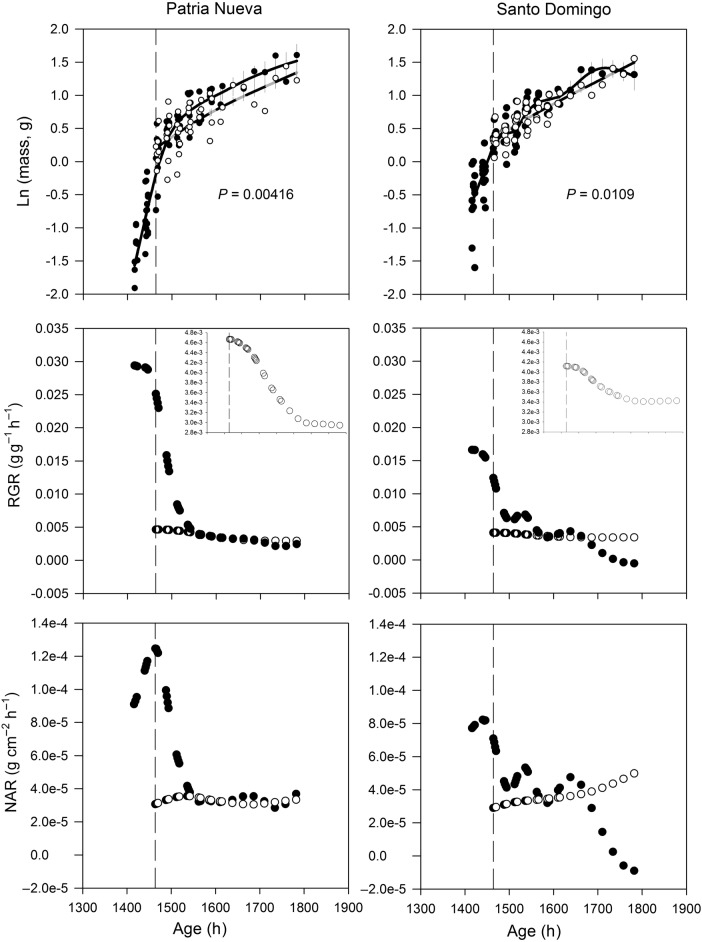
The time-course of shoot dry mass, RGR and NAR in two ecotypes of *D. stramonium* (PN and SD). Filled circles represent plants grown without defoliation (continuous line) and open circles represent defoliated plants (30 % of leaf removed) (dashed grey line). Standard errors are plotted in each fitted line. The dashed vertical line indicates the onset of the defoliation treatment. The inset graphs are a zoom of the time trajectory in the defoliated environment.

The ontogenetic trend in plant dry mass of defoliated plants began to diverge from that of undefoliated controls once defoliation occurred, and surpassed their values at the end of the experiment, mainly in the SD ecotype (Fig. 2). Relative growth rate decreased during plant development in the control condition. The maximum RGR of the PN ecotype was ∼29.4 mg g^−1^ h^−1^ at the beginning of the experiment and then decreased to around 2.45 mg g^−1^ h^−1^ at the end of the experiment (Fig. 2). However, a different trend was observed in the SD ecotype: the maximum RGR decreased by ∼29 % in relation to PN; it was 16.6 mg g^−1^ h^−1^ at the beginning of the experiment and decreased with a complex growth trajectory to zero at the end of the experiment, although two sudden increases were observed between 1494–1536 and 1586–1638 h of age (Fig. 2). The RGR values of the PN-defoliated plants immediately decreased to 4.7 mg g^−1^ h^−1^ (an 81 % decrease with respect to the same value at the time of defoliation in the control plants, i.e. 25.1 mg g^−1^ h^−1^) and slowly decreased to around 2.9 mg g^−1^ h^−1^ at the end of the experiment. As a result, the RGR of defoliated plants reached the RGR values of control plants with a marginal increase in the final phase of the experiment (Fig. 2). At the time of defoliation, the RGR values of the SD-defoliated plants immediately decreased to 4.1 mg g^−1^ h^−1^ (a 67 % decrease with respect to the same value at the time of defoliation in the control plants, i.e. 12.4 mg g^−1^ h^−1^) and remained almost constant, slightly decreasing to around 3.4 mg g^−1^ h^−1^ at the end of the experiment. As a result, the RGR of defoliated plants remained marginally lower than the control plants up to hour 1586, when defoliated plants reached the RGR values of control plants, producing an increase with respect to the values observed in the control plants at the end of the experiment (Fig. 2).

The NAR presented the same behaviour as RGR in the two ecotypes (Fig. 2). No errors are shown because NAR was estimated as a function of the means of RGR, SLA and LWR. A decrease in NAR was observed in the control condition for both ecotypes, the maximum (and minimum) values for the PN and SD ecotype were 1.2 × 10^−4^ g cm^−2^ h^−1^ (3.6 × 10^−5^ g cm^−2^ h^−1^), and 8.2 × 10^−5^ g cm^−2^ h^−1^ (−8.83 × 10^−6^ g cm^−2^ h^−1^), respectively. When the defoliation occurred, NAR values decreased to ∼3 × 10^−5^ g cm^−2^ h^−1^ in the two ecotypes (a 75 % decrease with respect to the same value at the time of defoliation in the control plants, i.e. 1.2 × 10^−4^ g cm^−2^ h^−1^). After that, NAR remained almost constant in the PN ecotype, but in the SD ecotype it increased slightly (5 × 10^−5^ g cm^−2^ h^−1^) and surpassed the values of control plants at the end.

**Figure 3.**
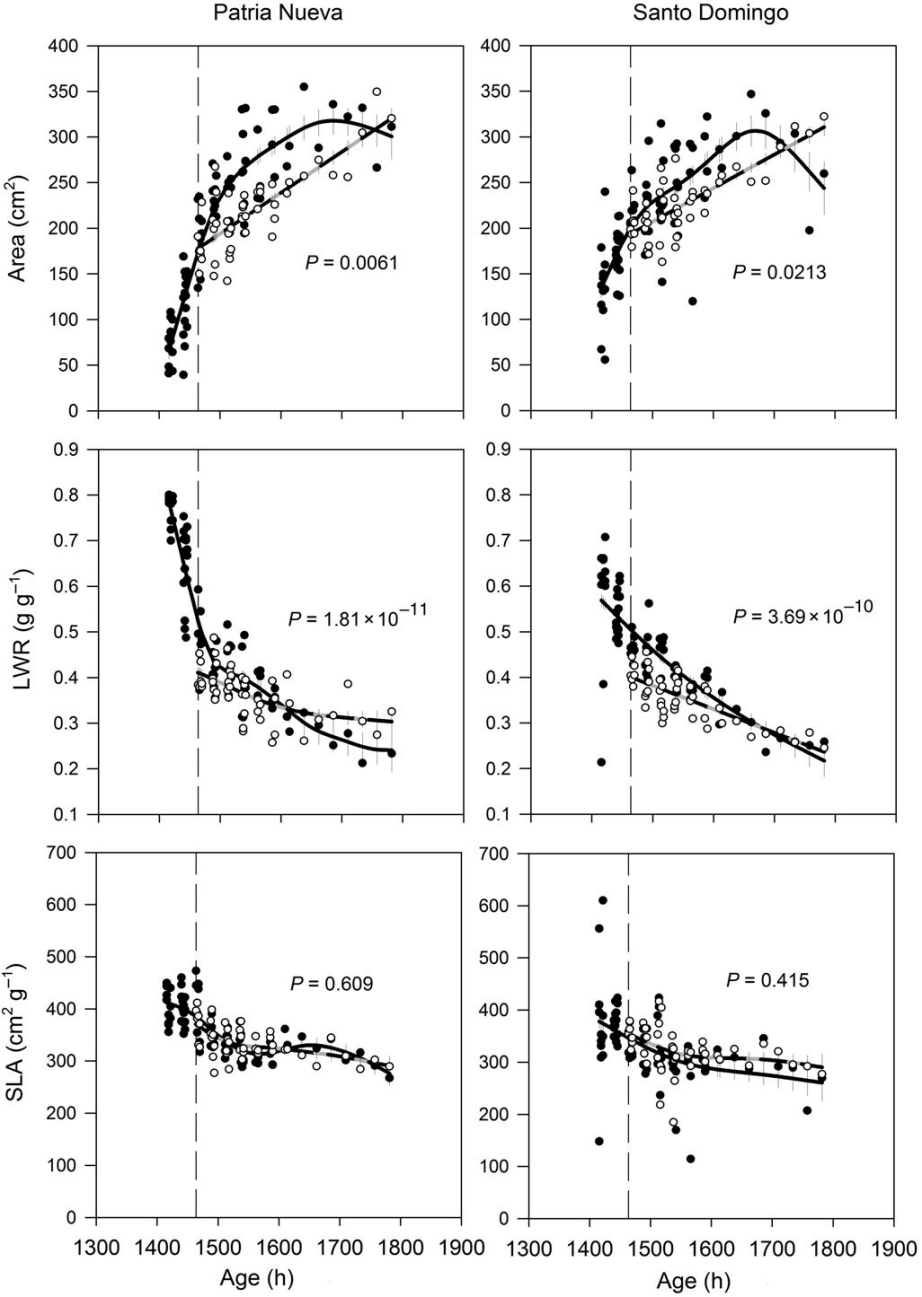
Area, LWR and SLA in two ecotypes of *D. stramonium* (PN and SD). Filled circles represent plants grown without defoliation (continuous line) and open circles represent defoliated plants (30 % of leaf removed) (dashed grey line). Standard errors are plotted in each fitted line. The dashed vertical line indicates the onset of the defoliation treatment.

In general, data showed evidence of ontogenetic phenotypic plasticity to defoliation in almost all traits measured; that is, the values for the defoliated series were statistically significantly different to the controls for the time frame of leaf area, shoot dry mass and LWR in the two ecotypes (Fig. 3). Specific leaf area did not express ontogenetic phenotypic plasticity to defoliation (Fig. 3). Traits displayed different patterns of ontogenetic phenotypic plasticity. Leaf area showed a monotonic increase in defoliated plants along time, re-establishing the trajectory of control plants and surpassing their values at the end, mainly in the SD ecotype. Leaf weight ratio decreased through time in control and defoliated plants of both ecotypes. When defoliation occurred, values of LWR immediately decreased to around 0.4 g g^−1^ in both ecotypes (a 21 % decrease with respect to the same value at the time of defoliation in control plants; i.e. ∼0.51 g g^−1^ in both ecotypes) then progressively decreased afterwards, resulting in higher values for defoliated plants at the end mainly in the PN ecotype (Fig. 3). The time-course of SLA was qualitatively the same in both ecotypes, decreasing from ∼412 cm^2^ g^−1^ to around 276 cm^2^ g^−1^ in the PN ecotype and from ∼378 cm^2^ g^−1^ to around 260 cm^2^ g^−1^ in the SD ecotype (Fig. 3).

**Figure 4.**
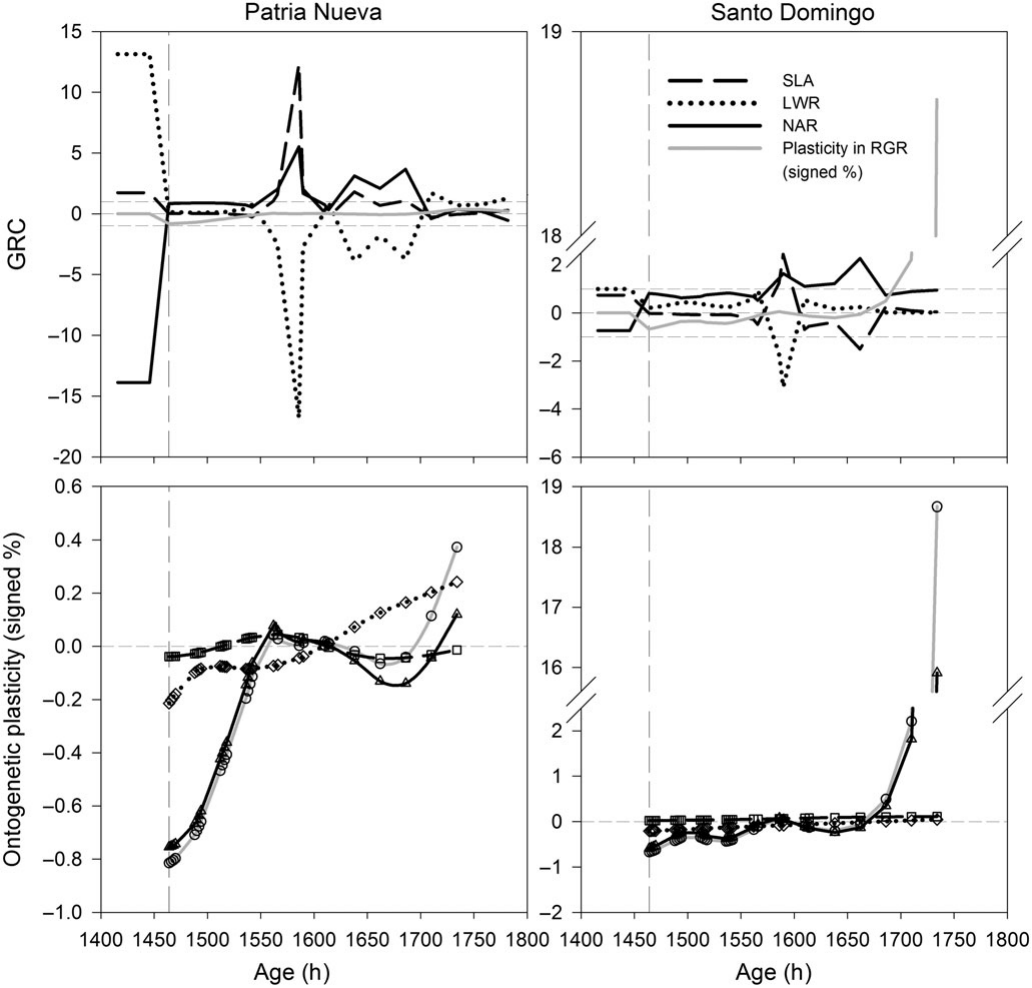
Time-course of GRCs and RGR in response to defoliation (vertical dashed line) in two ecotypes of *D. stramonium* (PN and SD). The higher panel shows the behaviour of plastic changes in RGR versus the changes in GRCs (horizontal dashed lines are reference values equal to 1 and −1). The lower panel is presented to show the ontogenetic plastic changes (used to estimate GRCs) in NAR, SLA and LWR underlying the plasticity in RGR.

The immediate RGR decrease due to defoliation is recovered after 98 h in the PN ecotype (1464–1562 h of age). In contrast, SD-defoliated plants equalled the RGR of control plants after 122 h (1464–1586 h of age; Fig. 4, upper and lower panels). Afterwards, defoliated RGRs remained almost constant with respect to controls in PN, but with a slight increase at the end of the experiment. Whereas in SD remained nearly constant with a large increase at the end of the experiment (Fig. 4, upper and lower panels). With this evidence, changes in RGR in response to defoliation could be classified into three main ontogenetic stages: the buffering, steady and overcompensatory states.

Growth response coefficient behaviour is linked to changes in RGR (Fig. 4, upper panels). Before defoliation, the RGR decrease was achieved by an ontogenetic decrease in NAR (GRC_NAR_ = −13.88, PN; GRC_NAR_ = −0.74, SD); that is, a strong decrease in NAR occurred with a decrease in RGR. This NAR decrease was accompanied by a positive counteracting LWR influence (GRC_LWR_ = 13.14, PN; GRC_LWR_ = 1, SD) and SLA (GRC_SLA_ = 1.74, PN; GRC_SLA_ = 0.74, SD), which prevented the RGR from falling even further. Immediately after defoliation (the buffering state), the proportional change in NAR almost equalled the proportional change in RGR; therefore, NAR was the most important trait increasing RGR in defoliated plants in both ecotypes. Nevertheless, the SD ecotype displayed an LWR contribution to the RGR increase in defoliated plants (Fig. 4, upper panels). Sizeable increases of defoliated-plant RGR (i.e. the overcompensatory state, values beyond control plants) can be achieved whenever the positive increase in a particular GRC cannot be counteracted by any other growth component. Interestingly, this overcompensation is achieved by a different GRC depending on the ecotype. For instance, the overcompensation at the end of the ontogeny observed in the RGR of the defoliated PN plants was achieved because the increase in LWR was not negatively influenced by the other growth components. At the same time, defoliated plants from the SD ecotype achieved overcompensation by NAR because other growth components did not counteract its effects.

**Figure 5.**
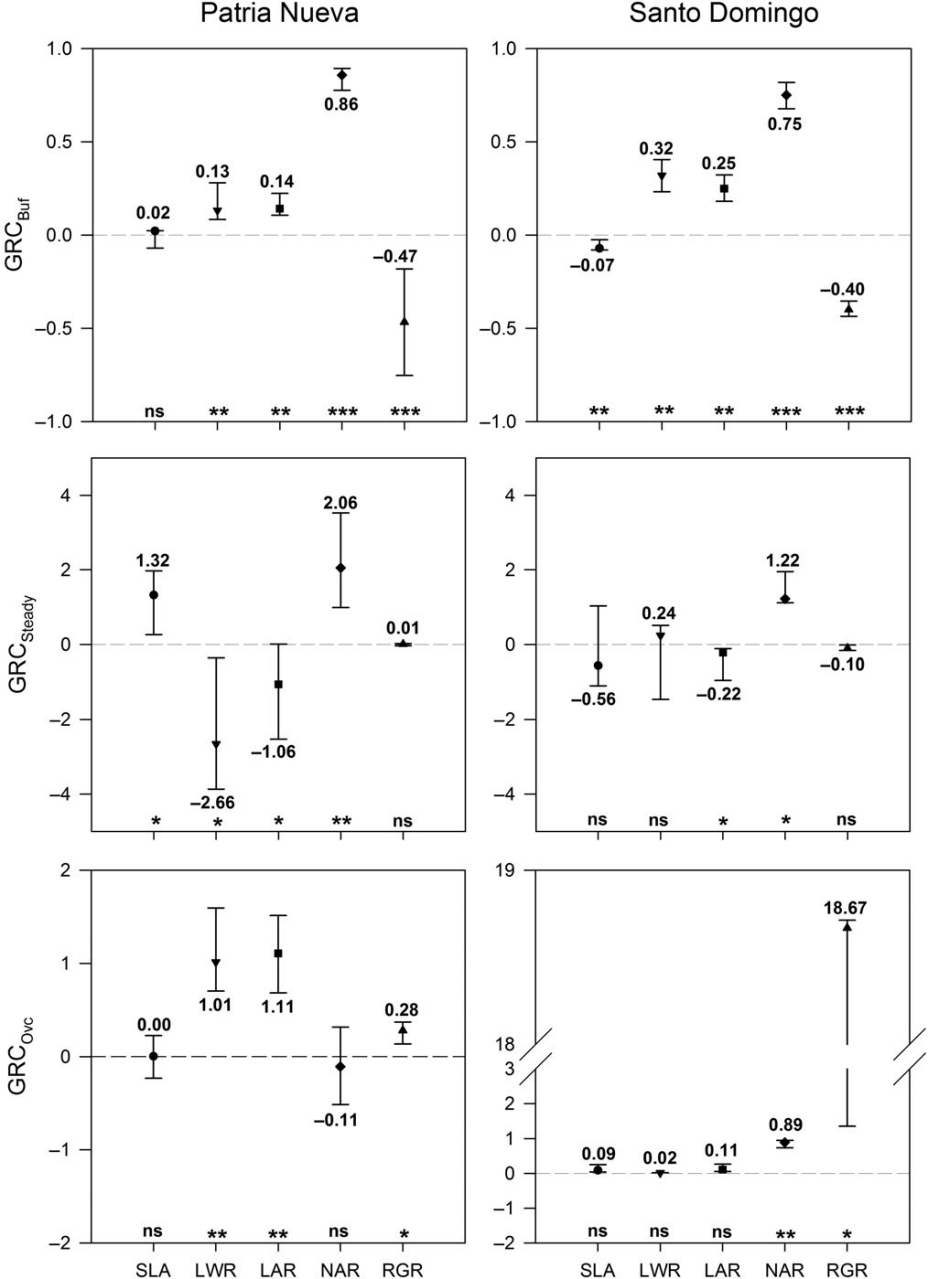
Error bars indicating the ontogenetic distribution by percentiles (first to third quartile) of GRCs of SLA, LWR and NAR in three main ontogenetic states of RGR (RGR_Buf_, buffering state; RGR_Ss_, steady state; RGR_ov,_ overcompensatory state) calculated for plants under different treatments (control and defoliated plants) in two ecotypes of *D. stramonium* (PN and SD). Plasticity in RGR (signed percentages, see Methods) are plotted for reference. Asterisks at the bottom of the panels indicate the significance level under the H_o_ hypothesis of GRC = 0. *P < 0.05; **P < 0.01; ***P < 0.0001. The printed values above or below the box plots give the median GRC values.

Net assimilation rate was the most important trait (PN; *T*_17_ = 76.5, *P* < 0.0001; SD; *T*_19_ = 95, *P* < 0.0001) contributing to RGR increase in defoliated plants throughout the buffering state, followed by LWR (PN; *T*_17_ = 59.5, *P* = 0.0016; SD; *T*_19_ = 76, *P* = 0.0006) in both ecotypes (Fig. 5). Leaf area ratio (the product of SLA and LWR) is more influenced by LWR. In the RGR steady state, a strong counteractive influence between NAR (*T*_8_ = 18, *P* = 0.0039) and LWR (*T*_8_ = −15, *P* = 0.0195; which mainly influenced LAR) produced no RGR differences (*T*_8_ = −1, *P* = 0.5273) between defoliated and control plants in PN (Fig. 5). In SD, this homeostatic transient RGR state (*T*_5_ = −6.5, *P* = 0.0625) was mainly related to a trade-off between NAR (*T*_5_ = 7.5, *P* = 0.0313) and SLA (*T*_5_ = −2.5, *P* = 0.3125; which mainly influenced LAR) (Fig. 5). Relative growth rate overcompensation (PN; *t*_4_ = 4.17, *P* = 0.0126; SD; *T*_4_ = 7.5, *P* = 0.0313) is mainly achieved because growth components did not trade-off, but it was mainly accomplished by different growth components depending on the ecotype: LWR in PN (*t*_4_ = 4.64, *P* = 0.0094) and NAR in SD (*t*_4_ = 13.58, *P* = 0.0027) (Fig. 5).

**Figure 6.**
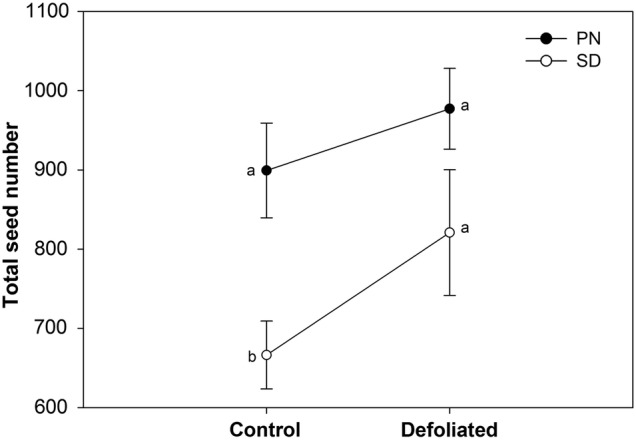
Norms of reactions of total seeds number for control and defoliated plants of two ecotypes of *Datura stramonium* (PN, Patria Nueva; SD, Santo Domingo) at the end of the experiment. Vertical bars represent standard errors of the mean. Different letters indicate significant differences between means at *P* < 0.05 (Tukey's HSD test).

Defoliated plants produced more seeds than controls (likelihood ratio test of treatment effect; *χ*^2^ = 261.38, d.f. = 1, *P* < 0.0001), demonstrating plasticity in fitness (Fig. 6). Ecotypes differ significantly in their mean production of seeds (*χ*^2^ = 697.30, d.f. = 1, *P* < 0.0001). The interaction between treatment and ecotype for the total number of seeds was significant (*χ*^2^ = 48.47, d.f. = 1, *P* < 0.0001), indicating ecotype variation in the tolerance capacity. Santo Domingo was the overtolerant ecotype; defoliated plants showed a 23 % increase in the number of seeds with respect to controls. In contrast, PN showed no fitness differences between environments, and then it was completely tolerant to defoliation (Fig. 6).

## Discussion

### Trade-off between RGR_max_ and tolerance to defoliation

As expected from our first hypothesis, we detected a trade-off between RGR_max_ and tolerance to defoliation. Santo Domingo was the most tolerant ecotype (i.e. overtolerant), leading to higher above-ground biomass and total number of seeds under defoliation at the end of the experiment. In addition, SD had the lowest RGR_max_. Therefore, the slow-growing ecotype showed a positive effect of defoliation on fitness in comparison with the fast-growing PN ecotype, which showed almost constant above-ground biomass and total number of seeds (i.e. complete tolerance) between control and defoliated plants at the end. Thus, the qualitative pattern of tolerance observed in this experimental study agrees with the natural pattern of tolerance to defoliation found in *D. stramonium* (e.g. Valverde *et al*. 2001, Fornoni *et al*. 2003). No differences in total plant biomass and increases in other fitness components in response to defoliation have been reported for grazing species in final ontogenetic stages (del-Val and Crawley 2005).

### Plasticity of RGR and tolerance to defoliation

Regarding our second hypothesis, greater tolerance is mediated by a higher plasticity in mechanistic traits. It was confirmed by the greater ontogenetic plasticity at the end of the experiment in overcompensatory growth expressed by the SD overtolerant ecotype (a 1866 % increase in RGR of defoliated plants with respect to controls) in comparison with the plasticity in overcompensatory growth of the PN ecotype (a 37 % increase in RGR of defoliated plants with respect to controls). This might be a case for adaptive plasticity, in which increasing plasticity in an underlying trait could increase fitness in one of a set of environments in which fitness was formerly similar, thereby increasing plasticity in fitness (Fig. 1A of Alpert and Simms 2002). If it is realized that RGR (in contrast to potential RGR) is positively related, via attainment of a larger size, to reproductive effort, survival and fecundity (e.g. Weaver and Cavers 1980; Aarssen and Taylor 1992; Crawley 1997), overcompensatory growth in the final stage of the ontogeny could constitute a selective advantage by helping plants to accumulate more biomass and produce more seeds in the presence of herbivores (e.g. *Themeda triandra*, Oesterheld and McNaughton 1988).

### Traits controlling ontogenetic plasticity of RGR in response to defoliation

Regarding the general question on what growth determinants are involved in the behavioural changes of RGR in response to defoliation, we have found that, throughout their ontogeny, defoliated plants of *D. stramonium* are capable of restoring, equalling or even overcompensating RGR compared with undefoliated plants. However, populations can differ in the importance of the component that controls the ontogenetic behaviour of RGR in response to defoliation.

#### In the buffering state

Progressive ontogenetic enhancements of NAR after defoliation contributed more to increasing RGR than enhancements of any other parameter. Increases in NAR after defoliation have been previously reported (Anten *et al.* 2003; Van Staalduinen and Anten 2005) and can be achieved through an increase in light intensity on the remaining leaves in grasses (Gold and Caldwell 1990; Senock *et al.* 1991; Van Staalduinen and Anten 2005) or by increased photosynthetic capacity (Nowak and Caldwell 1984; Zhao *et al.* 2008). However, other studies found no NAR increases (Van Staalduinen *et al.* 2010; Dobarro *et al.* 2012) after defoliation, but increases of leaf nitrogen concentration, a trait highly correlated with photosynthesis (Nowak and Caldwell 1984), suggesting that higher respiratory rates counteracted the increments in photosynthesis. This study did not measure photosynthetic capacity or levels of light intensity across the canopy. More studies are needed to evaluate the possibility of an increased photosynthetic capacity after defoliation in *D. stramonium*, as reported for other herbs (Zhao *et al.* 2008). Specific leaf area did not contribute to restoring the RGR after defoliation in both ecotypes (cf. Oesterheld and McNaughton 1988, 1991).

#### In the steady state of growth

A large trade-off between NAR and LAR produces no RGR differences between defoliated and control plants. However, ecotypes differed in the growth components that influence LAR. Leaf weight ratio produces a large trade-off with NAR in PN and SLA trade-off with NAR in SD.

#### In the overcompensatory state of growth

Leaf weight ratio was the most important trait in PN and NAR in SD. Net assimilation rate has been shown to be the most important trait conferring RGR overcompensation in *T. triandra* (Oesterheld and McNaughton 1988, 1991). However, *Dactylis glomerata* overcompensated for RGR after defoliation mainly by slowing down leaf senescence and, to a lesser extent, by increasing LWR (Dobarro *et al*. 2012). Differences in the growth component favouring overcompensatory growth may be due to a selection of one or more traits underlying RGR (Poorter 2002) in the different habitats of the ecotypes analysed. We predicted that NAR would be the trait involved in overcompensatory growth states, because of its very plastic nature, in contrast to perhaps more costly changes in biomass allocation or morphology. Considering that the PN ecotype's habitat receives very low mean annual precipitation (Table 1), it is possible that the strong water deficit in PN has favoured LWR over NAR, since decreases in photosynthetic ability due to a decrease in CO_2_ assimilation can be possible even with small water deficits (Kaiser 1987). Similarly, in the understory palm *Chamaedoera elegans* (Anten *et al*. 2003) and several grass species (Van Staalduinen and Anten 2005; Van Staalduinen *et al.* 2010; Dobarro *et al.* 2012), defoliated plants allocated considerable more mass to the production of leaf laminas than control plants, favouring increases in LAR. Leaf weight ratio changes can be seen as adaptive in response to defoliation, enabling more CO_2_ and light to be captured after a decrease in the available photosynthetic area. Here we assume that leaves (the organ involved directly in the acquisition of above-ground resources) have a priority over light and CO_2_, demanding and limiting more of the available photosynthates (Poorter and Nagel 2000).

A higher overcompensatory growth can be observed in adult plants when each component (NAR, SLA and LWR) does not negatively affect others, so that changes in these components are not largely cancelled out. It is intriguing as to why this overcompensation is expressed at the final stage of the ontogeny in contrast to the homeostatic effect observed in early stages of the ontogenetic trajectory, in which at least one growth component experiences a large trade-off to maintain the RGR. We can tentatively conclude that this instance of overcompensation in *D. stramonium* could mainly be related to buffer the differences in total reproductive output between defoliated and control plants (see above) in the SD ecotype. It is possible that the excess of photoassimilates in this ecotype produced by a higher NAR in defoliated plants could be due to buffering differences in total biomass and, additionally, could lead to increasing seed output.

## Conclusions

Using a high level of temporal resolution of growth analysis we were able to estimate the RGR_max_, and at an intra-specific scale to detect a trade-off between the ability to grow in benign environmental conditions and the ability to tolerate resource limitation due to defoliation. In addition, the fast-growing ecotype (PN) showed a higher diminished RGR after defoliation, but also exhibited the lowest increase later. The opposite was observed in the slow-growing ecotype (SD), which was least affected early but showed the highest compensation later, not only in RGR but also in fitness. This study supports the hypothesis of Hilbert *et al*. (1981) that compensatory growth is most likely when undefoliated plants are growing at low rates because the amount of RGR change required for defoliated plants to equal the productivity of undefoliated plants is lower since RGR of undefoliated plants decreases. At the same time, if plants are growing close to their maximum capacity (i.e. RGR close to RGR_max_), defoliation cannot increase RGR, and then compensation is unlikely (Hilbert *et al.* 1981; Oesterheld and McNaughton 1991). In line with Oesterheld and McNaughton (1988), this study showed that the ability of a genotype to tolerate defoliation will depend on the magnitude of the initial reduction in RGR, how fast the genotype reaches the equality point and how much RGR increases after that. More studies using within- and between-species comparisons are necessary to prove this prediction.

## Sources of Funding

This study was supported by the Consejo Nacional de Ciencia y Tecnología (CONACyT) grant project 81490 to J.N.-F.

## Contributions by the Authors

I.D.C. and J.N.-F conceived and designed the experiment, I.D.C., J.N.-F. and R.T.-L performed the experiment and collected the data, I.D.C. analysed the data and I.D.C. and J.N.-F. wrote the paper.

## Conflict of Interest Statement

None declared.

## Appendix

The following routine in R, using the MGCV package (Wood 2011), was used to evaluate the differences between control and defoliated plants (see Methods):

# loading data

dat<-read.table(file="C:/…", header=T)

# Defoliated as factor

dat$Defoliated<-as.factor(dat$Defoliated)

# Load GAM and GAMM package

library(mgcv)

# Multifactorial GAM with Damage and Hours interaction

y.fit <- gam(y ∼ Defoliated + s(Hours, by=Defoliated), data = dat, family=gaussian, bs= “cr”)

summary(y.fit)
